# Evaluation of Pediatric Chest Pain in the ED: Impact of the COVID-19 Pandemic

**DOI:** 10.7759/cureus.61829

**Published:** 2024-06-06

**Authors:** Noah Kondamudi, Rucha Patki, Majo Joseph

**Affiliations:** 1 Pediatrics, The Brooklyn Hospital Center, Brooklyn, USA; 2 Pediatrics, Icahn School of Medicine at Mount Sinai, New York, USA; 3 Emergency Medicine, The Brooklyn Hospital Center, Brooklyn, USA

**Keywords:** pediatric emergency department (ped), cardiac troponin, covid-19, lab workup, chest pain

## Abstract

Introduction

Chest pain is a common presenting complaint among children presenting to the ED, and serious underlying illnesses are found in only a small minority of cases. Due to the lack of established guidelines, the workup of these patients is institution or physician-dependent. Unlike adults with chest pain, workup among children tends to be minimal unless elements in the history and physical exam trigger it. We hypothesize that the emergence of COVID-19-related multisystem inflammatory syndrome in children (MISC) may have increased variability in how these patients are evaluated in the ED.

Objective

To determine if there has been a change in the approach to evaluating children presenting to the ED with chest pain since the emergence of the COVID-19 pandemic.

Materials and methods

This retrospective cohort study was conducted in a pediatric emergency department (PED) at a 400-bed urban academic community hospital. Medical records of children <21 years old who presented to the ED with chest pain from January to July in both 2019 and 2020 were reviewed. Patients with chest pain due to acute asthma exacerbations were excluded. Data about patient demographics, the number and types of tests utilized, and clinical management, including therapies and disposition, were collected. The subjects seen during 2019 were labeled as the ‘pre-pandemic group’ and those seen in 2020 as the ‘pandemic group’. The number and type of tests utilized, therapeutic interventions, and disposition during the two study periods were subjected to analyses.

Results

Of the 180 patients evaluated for chest pain, 32 were excluded due to physician-diagnosed asthma-related chest pain. The study thus included the remaining 148 patients. There was no statistical association between the pre-pandemic and pandemic groups for presenting features of fever, cough, tachycardia, tachypnea, time of presentation to the ED, electrocardiogram (EKG) performance, and chest X-ray. However, the pandemic group showed a statistically significant increase in lab tests and hospitalizations compared to the pre-pandemic group. There was a statistically significant increase in the performance of complete blood counts (CBC), C-reactive protein (CRP), lactic dehydrogenase (LDH), serum ferritin, creatinine kinase-MB (CK-MB), troponin, B-natriuretic peptide (BNP), and D-dimers.

Conclusion

Since the onset of the COVID-19 pandemic, there has been a trend toward more extensive lab workups for patients presenting with acute chest pain in the ED.

## Introduction

Chest pain is a common presenting complaint among children in the ED [1]. A cardiac etiology is found in only a small minority of cases [2-4]. Non-cardiac chest pain is the most common cause, with musculoskeletal pain accounting for 15-30% of these cases [5]. There is wide variation in clinical practice for managing acute chest pain in the ED [6]. The history and physical examination are instrumental in deciding whether further evaluation with tests is needed. Arrhythmias and myopericarditis account for most cases of cardiac chest pain among pediatric patients [7-10]. Elevated cardiac biomarkers are not always present in pediatric patients but, when elevated, are suspicious of a serious cardiac etiology [11,12]. During the COVID-19 pandemic, adult patients presenting to the ED, even with non-specific symptoms, were found to have elevated troponin levels and many had significant cardiac dysfunction [13]. Evaluation of cardiac biomarkers became a recommended part of the workup among adult patients [14]. Clinical presentation of COVID-19 varies widely among children [15]. The new SARS-CoV-2 related entity, multisystem inflammatory response syndrome in children (MIS-C), can result in cardiac involvement, and affected children may present with chest pain to the ED [16,17]. Elevated cardiac biomarkers were reported among children admitted for myocarditis with MIS-C [18]. The emergence of this new entity may have exacerbated the variability in testing these patients in the ED. We hypothesize that the frequency of diagnostic testing has increased for children presenting to the ED with chest pain. The objective of this study is to determine if there has been a change in the evaluation approach of children presenting to the ED with chest pain since the emergence of the COVID-19 pandemic.

## Materials and methods

A retrospective cohort study was conducted in a dedicated pediatric emergency department (PED) at a 400-bed urban academic community hospital. Medical records of all children under 21 years old seen in the ED with a chief complaint of chest pain between January 1 and July 31, 2019 (pre-pandemic period), and between January 1 and July 31, 2020 (pandemic period), were identified from the hospital's ED log. Patients with chest pain attributed to acute asthma attacks that resolved after treatment were excluded from the analysis. The identified medical records were individually reviewed to abstract data about patient demographics of age, race, and gender. Information about the time evaluated in the ED, lab tests performed, imaging studies, and disposition was collected from these records.

Statistical tests were conducted using SPSS for Windows Version 26. Frequencies were expressed as percentages, and the chi-square test was used to compare various categorical variables. Statistical significance was set at a p-value of <0.05.

## Results

Of the 180 patients evaluated for chest pain during the study period, 32 were excluded because the chest pain was attributed to physician-diagnosed asthma. The remaining 148 patients are the study subjects. The majority of these patients were female (n=92, 62%), over 13 years old (n=87, 59%), and of African American race (n=95, 64%). Most patients were seen during the 3 PM-12 midnight shift (n=71, 47%) compared to the morning or overnight shifts. In addition to chest pain, the other predominant symptoms were cough (n=18, 12%) and fever (n=5, 3%). Tachycardia on presentation was noted in 13 (9%) and tachypnea in five (3%) patients. An EKG was performed on 111 patients (75%), while only 49 (33%) had a chest X-ray. Lab testing/workup was obtained in 16 (11%) patients. The most common discharge diagnosis was musculoskeletal chest pain in 70 patients (47%) followed by non-specific chest pain in 49 patients (33%). Pneumonia was diagnosed in three patients (2%) (Table 1).

**Table 1:** Diagnoses for patients with chest pain.

Diagnosis	N	Percent
Musculoskeletal chest pain	70	47
Non-specific chest pain	49	33
Gastroesophageal reflux disease	11	7
Viral illness	8	5.5
Anxiety	4	3.5
Pneumonia	3	2
COVID-19	3	2
Total	148	100

The pre-pandemic group included 92 patients and the pandemic group had 56 patients. There was a statistically significant association between the older age group and visits during the pandemic compared to the pre-pandemic period, but no association with gender. A statistically significant association of the African American population was seen during the pandemic compared to the pre-pandemic period relative to other ethnicities. There was no statistical significance between the pre-pandemic and pandemic groups for presenting features of fever, cough, tachycardia, tachypnea, and time of presentation to the ED. There was also no significant association between performing an EKG or CXR between the two periods. However, there was a statistically significant association between performing lab tests and hospitalization in the pandemic group compared to the pre-pandemic group (Table 2). When analyzed for type of tests, there was a statistically significant increase in the performance of complete blood counts (CBC), C-reactive protein (CRP), lactic dehydrogenase (LDH), serum ferritin, creatinine kinase-MB (CK-MB), troponin, and D-dimers (Table 3). COVID PCR was performed on 45 of the 56 patients in the pandemic group, yielding three positive results that required hospitalization.

**Table 2:** Patient demographics and clinical presentation. EKG: Electrocardiogram; CXR: Chest X-ray.

Variables	Pre-pandemic	Pandemic	P-value
	N (%)	N (%)	
Age in years			<0.02
0 to 5	6 (7%)	0 (0%)	
6 to 12	36 (39%)	19 (34%)	
13 to 18	35 (38%)	28 (50%)	
18 to 20	15 (16%)	9 (16%)	
Gender			0.22
Female	35 (57%)	57 (66%)	
Male	27 (44%)	29 (34%)	
Ethnicity			<0.001
Black	48 (52%)	47 (84%)	
Hispanic	9 (10%)	5 (9%)	
White	7 (8%)	1 (2%)	
Asian	28 (30%)	3 (5%)	
Time seen in ED			0.09
7 AM-3 PM	32 (35%)	27 (46%)	
3 PM-12 MN	47 (51%)	24 (34%)	
12MN-7 AM	13 (14%)	5 (28%)	
Fever at presentation			0.78
Yes	4 (4%)	3 (5%)	
No	87 (96%)	53 (95%)	
Cough at presentation			0.53
Yes	10 (11%)	8 (14%)	
No	82 (89%)	48 (86%)	
Tachycardia			0.22
Yes	6 (6%)	7 (12%)	
No	85 (94%)	49 (88%)	
Tachypnea			0.29
Yes	2 (2%)	3 (5%)	
No	90 (98%)	53 (95%)	
Overall Lab Workup			0.0001
Yes	3 ( 3%)	13 (23%)	
No	89 (97%)	43 (77%)	
EKG			0.69
Yes	70 (76%)	41 (73%)	
No	22 (24%)	15 (27%)	
CXR			0.21
Yes	27 (30%)	22 (39%)	
No	65 (71%)	34 (61%)	
Hospitalization			<0.02
Yes	1 (1%)	5 (9%)	
No	91 (99%)	51 (91%)	

**Table 3:** Types of lab tests.

Lab Tests Performed	Pre-pandemic	Pandemic	P-value
	N (%)	N (%)	
Complete blood count			<0.01*
Yes	3 (3%)	12 (21%)	
No	89 (97%)	44 (79%)	
C-reactive protein			<0.01*
Yes	1 (1%)	6 (11%)	
No	91 (99%)	50 (89%)	
Lactic dehydrogenase			<0.003*
Yes	0 (0%)	6 (11%)	
No	92 (100%)	50 (89%)	
Ferritin			<0.007*
Yes	0 (0%)	5 (9%)	
No	92 (100%)	51 (91%)	
Creatinine kinase-MB			<0.001*
Yes	1 (1%)	9 (16%)	
No	91 (99%)	47 (84%)	
Troponin			<0.001*
Yes	3 (3%)	14 (23%)	
No	87 (97%)	43 (77%)	
Brain natriuretic peptide			0.13
Yes	2 (2%)	4 (7%)	
No	90 (98%)	52 (93%)	
D-dimers			<0.0001*
Yes	0 (0%)	10 (18%)	
No	92 (100%)	46 (82%)	

## Discussion

This study evaluated changes in the diagnostic evaluation and resource utilization for chest pain among pediatric patients presenting to the ED during the COVID-19 pandemic. Chest pain is a common complaint in pediatric ED visits and is frequently due to musculoskeletal issues rather than more serious cardiac or pulmonary causes. History is a key element in evaluation, and physical examination findings direct the workup, with most cases not requiring any tests. Associated symptoms such as syncope or exercise-induced chest pain, and a family history of sudden cardiac death, often increase the suspicion of a cardiac cause [19]. Cardiac biomarkers are typically only indicated for pediatric patients if there is an ongoing concern for myocarditis, pericarditis, or other serious cardiac pathology. Our study demonstrated a significant increase in laboratory testing/workup after the emergence of the COVID-19 pandemic (23.2% in 2020 vs. 3.2% in 2019). Lab studies performed included CBC, CMP, serum troponin, BNP, D-dimer, and CK-MB levels. COVID-19 infections, initially described in early 2020, predominantly impacted the adult population with pulmonary manifestations such as pneumonia and pulmonary embolism [20,21]. Limited understanding of COVID-19's clinical course among children posed new questions regarding the diagnostic evaluation of acute chest pain. As the pandemic evolved, multiple cases of MIS-C were reported [22,23], with new clinical patterns of myocardial injury emerging among pediatric patients with SARS-CoV-2 infection [24-26].

The American Academy of Pediatrics (AAP) published interim guidelines in February 2021 for the diagnosis of MIS-C, emphasizing diagnostic testing for children presenting with prolonged symptoms such as fever, cough, and gastrointestinal symptoms (abdominal pain, vomiting, diarrhea) accompanied by evidence of organ dysfunction [27]. Only 5% (n=3) of patients had fever and 14% (n=8) had cough concurrently with chest pain upon arrival at the ED.

The clinical presentation of COVID-19 among children varied widely, with chest pain being one of the symptoms often associated with cardiac involvement [28]. In a case series of 20 children with MIS-C by Grimaud M et al., acute myocarditis with elevated inflammatory biomarkers was observed in all patients [17]. Troponin was the most commonly ordered test for patients with chest pain (23.6%), followed by D-dimer (16%), CK-MB (16%), and CRP (10.7%). We also noted an increase in hospitalizations (9% vs. 1%), three of whom were COVID-19 positive. In a study of 315 hospitalized children with laboratory-confirmed SARS-CoV-2 infection by Fernandez DM et al., elevated biomarkers were associated with disease severity [29].

This study identifies a trend in changing management paradigms for chest pain among children presenting to the ED. Limitations of the study include the small sample size, being limited to a single institution, and the early period after the onset of the pandemic. These trends may change over time as more data becomes available and patterns of COVID-19 illness emerge.

## Conclusions

There has been an emerging trend of increased lab testing and workups since the onset of the COVID-19 pandemic for patients presenting with acute chest pain to the ED. This suggests a need to update guidelines for the appropriate diagnostic workup of acute chest pain in children in the post-COVID-19 era, given the concerning variations in management approaches and resource utilization. Further studies are warranted to evaluate chest pain using standardized diagnostic tools in pediatric patients.
